# IκBα beyond the NF-kB dogma

**DOI:** 10.18632/oncotarget.1325

**Published:** 2013-09-27

**Authors:** Maria Carmen Mulero, Anna Bigas, Lluís Espinosa

**Affiliations:** Program in Cancer Research, Institut Hospital Mar d'Investigacions Mèdiques (IMIM), Barcelona, Spain

The NF-κB pathway participates in a myriad of processes including cell differentiation, proliferation, survival, and regulation of the immune response. In mammals there are five NF-κB proteins: p65/RelA, p50, RelB, cRel and p52. All these proteins can form dimers to generate more than a dozen of possible combinations. Different NF-κB dimers bind the DNA at specific κB sites, acting as activators or repressors of gene transcription. However, this is only an example of how complex the NF-κB pathway is, since it also involves several post-translational modifications of upstream and downstream elements and multiple negative and positive regulatory loops.

IκB proteins, including IκBα, IκBβ and IκBε constitute the canonical inhibitors of the NF-κB pathway. However p105 and p100 proteins, the precursors of p50 and p52, contain a C-terminal end that is functionally comparable to IκB (this is the reason they are also called IκBγ and IκBδ, respectively).

In unstimulated cells, NF-κB dimers reside in the cytoplasm bound to IκB proteins. Upon stimulation, IκBs become phosphorylated, subsequently polyubiquitinated and degraded by the proteasome. Degradation of IκB leads to the release of the NF-κB factors that then translocate to the nucleus to activate transcription of specific genes including IκBα itself. Termination of the transcriptional response primarily depends on IκBα resynthesis that is required not only for retaining NF-κB in the cytoplasm but also for removing active NF-κB dimers from the DNA [1]. Because this mechanism implies that IκBα continuously shuttles from the cytoplasm to the nucleus to maintain the cells in a silent NF-κB state [2], it is not so surprising that IκBα has been found associated with other proteins that are primarily nuclear such as histone deacetylases (HDACs) or nuclear corepressors [3, 4]. Now, we have demonstrated that IκBα is predominantly nuclear in primary keratinocytes where it binds the chromatin at specific genes promoters associated with their transcriptional repression [5]. Mechanistically, we found that IκBα facilitates the association of the Polycomb Repressive Complex 2 (PRC2) to specific promoters and supply PRC2 with the capacity to respond to TNFα stimulation, thus establishing an unexpected link between inflammatory signals and skin homeostasis. This result is particularly relevant since to date it is still not clear how PRC2 is recruited to the chromatin in mammals, unlike in flies where specific PRC2 recognition DNA sequences have been identified. The physiologic relevance of IκB and Polycomb (Pc) association has been validated in Drosophila using compose mutants of Cactus, the homolog of IκBα in flies, and Pc, which in fact indicates the elevated conservation of this function during evolution.

The identification of IκBα as a chromatin interacting protein apparently contradicts the notion that IκBα cannot bind the DNA. However, it is not just IκBα but a SUMOylated and phosphorylated form of the protein that binds the chromatin, suggestive of structural changes that affect the biochemistry of the protein including its capacity to bind NF-κB or the chromatin in a mutually exclusive manner. Another member of the family, IκBβ, also binds the chromatin but only when it is hypophosphorylated [6], suggesting that post-translational modifications of the NF-κB elements link this pathway with specific cellular functions. This is also the case of IKKα and NEMO that exert additional NF-κB-related but independent functions such as cell cycle control and DNA-damage repair, respectively [7, 8]. However, which are the modifications that regulate alternative IKKα and NEMO functions is not completely understood.

**Figure 1:**
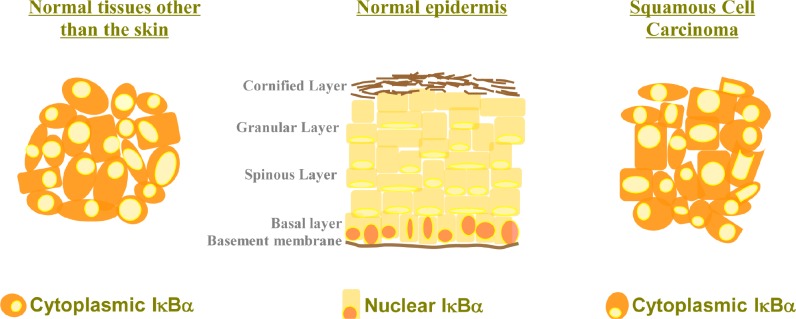
Cytoplasmic localization of IκBα in the skin is associated with tumorigenesis

Detection of cytoplasmic IκBα has never been considered as pathologic in any tissue, however this seems to be the case in the skin (Fig. 1). In Mulero et al we found that nuclear exclusion and cytoplasmic accumulation of IκBα strongly associated with progression of Squamous Cell Carcinoma (SCC) in patient samples. Although this is not addressed in the study, it is plausible that this is not cytoplasmic accumulation of conventional IκBα but of SUMOylated IκBα what exerts pro-tumorigenic functions, likely by relieving the nucleus from transcriptional regulators. Further studies should be performed to validate this idea. Why this is observed in the skin but not in other tissues can be basically a matter of levels of either phosphatases and/or desumoylases, which will be deciphered in the near future. However, this does not exclude that low amounts of SUMOylated IκBα exert comparable functions in cells other than the keratinocytes, which will be also investigated.

IκBα is localized in the cytoplasm of most cell types where it plays a major role as a negative regulator of the NF-κB signaling pathway. However, IκBα is detected in the nucleus of basal layer keratinocytes, whereas loss of nuclear IκBα and its cytoplasmic accumulation is a hallmark of human Squamous Cell Carcinoma.

In conclusion, the identification of SUMOylated and phosphorylated IκBα as a spinoff of IκBα that plays unexpected but important physiologic function opens a whole field of research and the possibility to re-interpret some old unexpected results with a novel and renewed perspective.
